# Follicle-Stimulating Hormone-Secreting Pituitary Adenoma Inducing Spontaneous Ovarian Hyperstimulation Syndrome, Treatment Using *In Vitro* Fertilization and Embryo Transfer: A Case Report

**DOI:** 10.3389/fendo.2021.621456

**Published:** 2021-06-24

**Authors:** Xiaofang Du, Wen Zhang, Xingling Wang, Xiaona Yu, Zhen Li, Yichun Guan

**Affiliations:** Reproductive Medicine Center, The Third Affiliated Hospital of Zhengzhou University, Zhengzhou, China

**Keywords:** FSH-secreting pituitary adenoma, IVF-ET, spontaneous ovarian hyperstimulation syndrome, transsphenoidal surgery, endocrine disorders

## Abstract

**Objective:**

To describe the management of a patient with a pituitary adenoma secreting follicle-stimulating hormone (FSH) associated with spontaneous ovarian hyperstimulation syndrome (sOHSS) who was treated with *in vitro* fertilization and embryo transfer (IVF-ET).

**Methods:**

We report a clinical case of a woman of reproductive age with menstrual irregularity, infertility and ovarian hyperstimulation due to recurrent pituitary adenoma secreting FSH, which persisted after transsphenoidal surgery.She underwent the diagnosis by magnetic resonance imaging (MRI) and laboratory tests,and finally she was treated with IVF-ET.

**Result(s):**

The patient was plagued by a recurrent pituitary adenoma for many years and tried various treatments. After complete transsphenoidal surgery, sOHSS decreased, as shown by a reduction in oestradiol levels and an improvement in the ultrasonography parameters; however, secondary amenorrhea occurred. Finally, pregnancy was achieved through IVF-ET and the symptoms of ovarian hyperstimulation were relieved.

**Conclusion(s):**

IVF-ET was found to be effective for the treatment of recurrent pituitary adenoma, thus representing a therapeutic option that should be taken into consideration in such cases.

## Introduction

Ovarian hyperstimulation syndrome (OHSS) occurs most frequently as an iatrogenic complication from the implementation of assisted reproduction technology (ART) of exogenous gonadotropin administration for ovulation induction. It manifests as enlarged ovaries, abdominal pain, bloating and ascites. More severe clinical manifestations include renal failure, respiratory distress syndrome and thromboembolism. However, spontaneous OHSS(sOHSS) is a rare condition that has been reported in patients who achieve natural pregnancy without ovulation stimulation, severe primary hypothyroidism and granulosa cell tumours (1–3).

Gonadotroph adenomas are usually clinically nonfunctioning or are silent and difficult to identify. Approximately 25.2% to 64% of clinically nonfunctional pituitary adenomas were confirmed by postoperative immunohistochemistry to be pituitary gonadotropin adenomas (4, 5). Clinically functioning gonadotroph adenomas are rarely known but can exhibit significant symptoms, including spontaneous ovarian hyperstimulation and abnormal menstrual cycles (i.e., amenorrhea and rare menstruation) in premenopausal women (6). Furthermore, gonadotroph adenomas can manifest as breast development, vaginal bleeding and enlarged ovaries in prepubertal women. To date, few cases of gonadotroph pituitary adenoma-induced OHSS have been reported (7, 8). This is concerning because their exact prevalence remains unknown despite their persistence and their recurrence having been commonly described in the literature (9).

This article is a clinical case report of a patient with sOHSS caused by recurrent FSH-secreting pituitary adenoma. The incidence rate and clinical characteristics of pituitary adenomas were identified by the characteristics of pituitary adenomas. Because ovarian function did not return to normal after surgery and there was no normal ovulation, we retrieved oocytes for IVF-ET and finally achieved pregnancy. This process provides a new way to clinically diagnose and treat this disease in the future.

## Materials and Methods

### Patient

A 27-year-old nulligravida was referred to the Reproductive Department of the Third Affiliated Hospital of Zhengzhou University for irregular menstruation and infertility. She was diagnosed with pituitary adenoma secreting FSH with sOHSS. The diagnosis was made based on serum hormone levels and cranial MRI;The patient underwent a transsphenoidal partial resection of the tumour and the histopathology and immunohistochemistry of tissue that confirmed the final diagnosis. According to the postoperative serum hormone levels and the recovery of ovarian function, the patient was subsequently undergone IVF-ET. Written informed consent was obtained from the individual(s) for the publication of any potentially identifiable images or data included in this article.

### Assays for Gonadotropins and Steroid Hormones

Serum hormone concentrations were measured by electrochemiluminescence using reagents from Roche Diagnostics (Cobas; Roche Diagnostics). Tumour markers were measured by chemiluminescent immunoassay using reagents from German Siemens (ADVIA Centaur CP;German Siemens).

## Results

This infertile woman presented to our center complaining of a 7-year history of menorrhagia and irregular menstruation. She underwent two laparoscopic bilateral ovarian cyst resections at other hospitals for treatment of multiple ovarian cysts, and histology confirmed that they were benign follicular cysts. However, after surgical treatment, the ovarian cyst recurred, and the menstrual cycle did not return to normal. On physical examination, she had a body weight of 64 kg, a body mass index of 22.4 kg/m2 and a blood pressure of 117/82 mmHg. At pelvic ultrasound, she presented markedly enlarged ovaries with multiple cysts which had regular walls and transonic content, similar to what found in ovarian hyperstimulation. However, there was neither ascitic fluid nor severe pelvic pain. The rest of the clinical examination was normal. And she denied other neurologic symptoms including headache and dysosmia. Her endocrinological profile was prolactin (PRL) 10.46ng/mL (reference range: <30ng/mL), thyroid−stimulating hormone(TSH) 1.29 μIU/ml (reference range: 0.38–4.34 μIU/ml), free T4 1.28ng/dl (reference range 0.81–1.89 ng/dl),and FSH 11.6 mIU/ml (reference range 3.85–21.51 mIU/ml).

**Figure 1:**
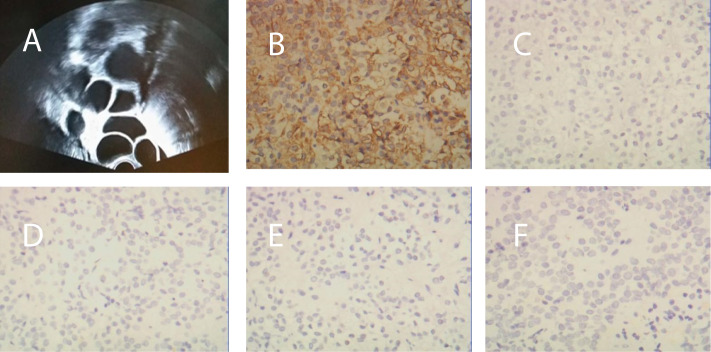
**(A)** Transvaginal ultrasonography before surgery revealed bilaterally enlarged ovaries with multiple follicles. **(B)** Immunohistochemical staining of the pituitary adenoma for FSH, **(C)** GH, **(D)** LH, **(E)** PRL, and **(F)** ACTH.

From May to September 2014, the patient was treated with oral contraceptives was injected with a gonadotropin-releasing hormone agonist to treat the multiple ovarian cysts and dysfunctional uterine bleeding. However, the treatment did not work. During this period, multiple measurements of serum sex hormone levels showed abnormalities, and Carbohydrate antigen 125(CA 125) and Carbohydrate antigen199(CA 199) were negative (**Table 1**). Subsequently, cyst aspiration was performed, and a small number of granulocytes, no oocytes and a mucus mass were found during the operation. Pathological examination showed that a small number of lymphocytes, neutrophils, phagocytes and well-differentiated epithelioid cells were found. Moreover, due to the elevated serum progesterone(P4), an adrenal ultrasound was performed to rule out adrenal diseases. Until November 2014, hormonal evaluation showed that the PRL level was elevated (52.48 ng/ml), therefore, a pituitary MRI was prescribed. The test showed the presence of a 25 x18 x17 mm pituitary adenoma that lacked normal pituitary morphology. Then, the patient underwent a transsphenoidal hypophysectomy. Immunohistochemical staining confirmed a pituitary adenoma that was markedly positive for FSH (90% of the adenomatous cells) and negative for all other anterior pituitary hormones (**Figure 1**). In 2017, despite undergoing fertility treatment at our hospital, the pituitary tumour recurred, and the patient underwent gamma knife treatment. Three months postoperation, the patient presented with amenorrhea and serum levels such that Luteinizing Hormone(LH) was suppressed (0.1 mIU/ml), FSH was normal and Testosterone(T) and PRL levels were elevated. Treatment proceeded with the administration of bromocriptine, ethinylestradiol and cyproterone acetate tablets for 2 months. Despite these treatment efforts, the patient still experienced amenorrhea and was subsequently advised to begin assisted reproductive therapy.

**Table 1:** Clinical characteristics and treatment of this patient.

Time	Menstrual cycle	Size of ovary*(cm)	Hormones (reference values)						Examinations	Medication	Treatment
			FSH (IU/L)Fp 3.5-11.5 mc 4.7-21.5 lp 1.7-7.7	LH (IU/L)Fp 2.4-12.6 mc 14-95.6 lp 1.0-11.4	E_2_ (ng/L)Fp 12.4-233 mc 41-398 lp 22.3-341	P4 (μg/L)Fp 0.2-1.5 mc 0.8-3.0 lp 1.7-27	T (nmol/L)(0.19-1.67)	PRL (μg/L)(4.79-23.3)			
2012.03	–	87	–	–	–	–	–	–			Ovarian cystectomy
2013.01	–	92	–	–	–	–	–	–			Ovarian cystectomy
2014.05	60	89	11.05	0.10	979.9	7.87	0.19	–	CA125 (-) CA199 (-)	E+P OC^1^	
2014.07	12	85	10.92	0.10	230.3	3.19	0.18	23.54		GnRH-a	
2014.08	11	97	10.77	0.10	975.2	6.67	0.27	30.98			Ovarian cyst puncture
2014.09	3	67	10.51	0.10	252.0	3.71	0.33	17.35	Adrenal ultrasonography		
2014.11	8	83	12.24	0.10	997.1	5.01	0.30	52.48	MRI		Transsphenoidal surgery
2017.03	17	91	13.50	0.20	1781.0	8.3	0.89	24.1	MRI		Gamma knife
2017.07	3	78	7.80	0.10	336.3	7.5	1.46	42.8		B OC^2^	
2017.08	5	65	3.00	0.10	62.9	0.5	0.46	0.5		B OC^2^	

*A major axis; fp, follicular phase; mc, mid-cycle; lp, luteal phase (in premenopausal women); E, ethinyl oestradiol; P, dydrogesterone; OC^1^, 0.15 mg desogestrel+30 μg ethinyl oestradiol; B, bromocriptine; OC^2^, 2 mg cyproterone acetate+0.035 mg ethinyloestradiol.

### Process of Assisted Reproduction

This patient tried to conceive naturally for 4 months but failed. After withdrawal of the therapies, the diameter of a follicle was less than 6 mm, she received the ovulation induction treatment at our center. 37.5 IU HMG was injected daily from cycle day 3 in a step-up protocol (**Table 2**). The dose of HMG was maintained and the follicular development was monitored by ultrasonogram continuously. When the follicular diameter reached 19mm, 10,000 U of hCG(Chorionic Gonadotrophin for Injection, Livzon Pharmaceutical Group) was injected to induce ovulation, after which she was instructed to have sexual intercourse within 24 hours. An ultrasound examination was performed 48 hours post hCG injection to observe follicle rupture, and oral dydrogesterone(Abbott Laboratories) 20 mg/d was administered for luteal phase support from the day of ovulation. Despite having attempted induced ovulation, the patient was unsuccessful in achieving pregnancy.

**Table 2:** Transition in follicular development during OI.

Menstrual cycle	Follicle size (mm)		HMG administration (IU)
	Rt	Lt	
5	6	6	0
9	12	11	150
11	15	11	300
12	17	13	375
14	19	14	525

IU international units.

The patient experienced withdrawal bleeding, and bilaterally enlarged ovaries with multiple cysts of which the largest diameter of 22mm were observed on day 12. Furthermore, a detailed hormonal examination showed elevated oestradiol(E_2_) levels (591.8 pg/ml) and progesterone(P4) levels (3.74 ng/ml), suppressed LH levels(<0.1 IU/L) and high-normal FSH values(12.78 IU/L)(**Table 3**). These results suggested that her pituitary tumour could recur again. Indeed, MRI showed a large recurrent tumor, measuring 22 x18 x16 mm. It was further determined that pituitary surgery failed to normalize her menstruation and ovulation cycle. Consequently, we planned a random oocyte retrieval for her. After the routine tests on her husband, including semen evaluation, sex hormone test and so on, then, the patient was prepared for oocyte retrieval by administering a recombinant HCG 250 μg (r-HCG, Ovidrel, Merck Serono, Germany) trigger two days prior to oocyte pick up. In total, 14 oocytes were retrieved from IVF, which produced 11 fertilized oocytes, while 4 high-quality cleavage-stage embryos were cryopreserved and the other embryos were performed blastocyst culture, but no blastocyst was formed.

**Table 3:** Transition in serum steroid hormone concentrations and follicular development during IVF.

Menstrual cycle	Follicle size(mm)		E2 (pg/mL)	FSH (IU/L)	LH (IU/L)	P4 (ng/mL)
	Rt	Lt				
12	12-21	15-22	591.8	12.78	<0.1	3.74

E_2_, estradiol; LH, luteinizing hormone; FSH, follicle stimulating hormone; P4, progesterone.

Postoperative endocrine investigations showed elevations in oestradiol and FSH(14.24 IU/L) and suppression of LH(<0.1 IU/L). Therefore, we suggest that she should be treated surgically to reduce the effect of pituitary tumours on embryo transfer and pregnancy. In April 2018, a transsphenoidal pituitary surgery was performed to completely remove the adenoma. Ultrasound evaluation after the operation revealed normal ovarian size and complete disappearance of the cysts. Immediate postoperative endocrine investigations showed reductions in E_2_, FSH and P4 and minimal changes in LH (**Table 4**). Last, the patient received estroprogestin therapy to normalize the amenorrhea that she had developed postoperation.

**Table 4:** Serum hormone levels before and after pituitary transsphenoidal surgery to remove FSH-secreting adenoma.

Time (d)	Reference range	Preoperative	Postoperative			
			2d	3d	4d	5d
LH (IU/L)	fp 2.12-10.89 mc 19.18-103.03 lp 1.20-12.86	0.28	0.25	0.20	0.20	0.20
FSH (IU/L)	fp <10 mc 4.54-30.34 lp 1.65-9.66	15.94	3.39	1.72	0.76	0.49
E_2_ (pg/mL)	fp 27-122 mc 95-433 lp 49-291	808.03	958.42	579.90	216.62	127.75
P4 (ng/mL)	fp 0.38-2.28 mc 0.93-2.23 lp 5.16-29.26	6.76	4.01	3.16	2.64	1.67
T (ng/mL)	0.10-0.75	0.56	0.73	0.81	0.49	0.52
COR (μg/dL)	4.0-22.3	19.77	–	–	–	10.69
PRL (ng/mL)	<30	46.62	16.30	12.71	13.06	11.55
GH (ng/mL)	<2	0.30	–	–	–	0.50
IGF-1 (ng/mL)	117-329	93	–	–	–	132
ACTH (pg/mL)	0-46	24.80	–	–	–	17.70

fp, follicular phase; mc, mid-cycle; lp, luteal phase (in premenopausal women); COR, cortisol; GH, growth hormone.

### Results of ART and Pregnancy

Two cleavage embryos were transferred in August 2018, the endometrium was prepared with hormone replacement treatment (HRT) due to pituitary amenorrhea. The serum level of β-hCG was 685 mIU/mL at day 14 after embryo transfer and clinical pregnancy was confirmed by the presence of a foetal heartbeat at 7 weeks after embryo transfer. Pregnancy proceeded without complications. At 39 weeks of gestation, cesarean section was induced and a healthy baby girl, weighing 3650g, was born.

## Discussion

Gonadotropin pituitary adenomas account for 80% - 90% of all nonfunctional pituitary adenomas and 40% - 50% of all pituitary macroadenomas. However, these adenomas do not present the characteristic symptoms of hormone hypersecretion, as there is often no clinical manifestation in the early stage. In most cases, it is not discovered until the tumour has enlarged and symptoms such as visual field defects and headaches appear (10). However, gonadotropin adenomas should not always be considered nonfunctional because these adenomas express and secrete active hormones, usually FSH, and cause typical clinical manifestations associated with excess hormone levels, such as menstrual irregularity, infertility and sOHSS, which can occur in premenopausal women (9, 11).

The pathogenesis of sOHSS remains unclear. However, the most plausible pathogenesis pathways are thought to be caused by mutations in the gonadotropin receptor gene. It has been found that T449A and D567N are two potential amino acid substitution sites (alanine substitution for threonine mutation); D567N is highly sensitive to thyrotropin(TSH) and has independent ligand activity. The affinity of the mutant FSH receptor protein for FSH, TSH and hCG is increased abnormally, which not only enhances the physiological effects of FSH, TSH and hCG but also stimulates pathological ovarian hyperstimulation (12, 13)

sOHSS due to a pituitary adenoma is a relatively rare disease with unspecific symptoms,and data about sOHSS are yet limited. We aimed to describe in detail the clinical and biochemical characteristics of reproductive age women with sOHSS caused by gonadotroph adenomas and to analyse the diagnosis and treatment.

### Epidemiology

Epidemiological studies of functional gonadotroph adenomas are limited (9), in part because of their rarity and nonspecific signs and symptoms, and in part because of an uncoupling of clinical presentation and pathologic evaluation of tumour specimens. Since 1995, Djerassi et al. first reported cases of spontaneous ovarian hyperstimulation caused by gonadotropin pituitary adenoma (14). Until now, only 37 cases have been reported in the literature. The incidence rate of the disease is not precisely known. A retrospective study analysed the clinical data of 171 women of reproductive age who underwent pituitary adenoma surgery. Among them, the incidence rate of OHSS in nonfunctioning pituitary adenomas was 2.9%, however it rises to 8.1% when the analysis of patients with a histologically established gonadotroph adenoma is restricted (15). However, the incidence of such cases is still not accurately assessed, due to the lack of detailed inspection, it may be underestimated.

### Clinical Manifestations and Auxiliary Examination

The current case aligns with previous studies such that the common clinical manifestations of this disease are menstrual abnormalities, including amenorrhea, oligomenorrhea and abnormal uterine bleeding. At the same time, it may be accompanied by bilateral ovarian multilocular cysts and abnormal enlargement of ovaries (16). While some patients will have abdominal pain, abdominal distention, lactation, headache, blurred vision and other symptoms, others will experience infertility. However, the clinical manifestations of hydrothorax and ascites caused by blood concentration and increased vascular permeability are not obvious. This is also a significant difference between sOHSS and iatrogenic OHSS (17).

Relative to healthy levels, the characteristic hormone levels of this type of case are as follows: E_2_ is significantly higher, LH is lower, FSH and PRL are higher and P is slightly higher or in the normal range. In the reported cases, the most common gonadotropin adenomas are FSH-secreting. Continuous FSH stimulation can induce multiple dominant follicle recruitment and increase E_2_ levels (18, 19). Because of the negative feedback mechanism of the hypothalamic pituitary ovarian axis, FSH may not increase significantly or remain in the normal range. However, normal FSH can still cause ovarian hyperstimulation, which may be related to the increased biological activity of FSH; alternatively, a pituitary adenoma may secrete other FSH subtypes, which then reach the threshold level of stimulating the sustained growth of follicles (20, 21). The decrease in LH levels may be due to the negative feedback inhibition of E_2_ and the compression of normal pituitary tissue and stalk by enlarged tumours. It may also be that the excessive secretion of FSH with abnormal structure inhibited the secretion of the gonadotropin releasing hormone(GnRH) receptor or hypothalamic GnRH and thereby inhibited the production of normal FSH and LH (22). We also noticed that the two-cell two-gonadotropin theory suggests that both FSH and LH are both required to stimulate the ovaries to secrete E_2_. LH stimulates theca cells to produce androgens, which are then transported to granulosa cells and aromatized to become E_2_ under the influence of FSH; if LH is absent, the E_2_ level should also be reduced (23). However, our case shows a high level of E_2_ and a low level of LH. This may mean that very low levels of LH are enough to produce E_2_, or there may be an alternative unknown cause. Our case also showed occasional elevated PRL, and previous studies also found that a small number of patients will have galactorrhoea or hyperprolactinemia. Prolactinemia is often an important piece of evidence in the identification of pituitary tumours. This may be caused by the pituitary stalk effect; that is, when the tumour compresses the pituitary stalk, the secretion of PRL inhibitor release is blocked, and the inhibition of hypothalamus secretion of PRL is weakened, resulting in an increase in PRL (20, 24). Moreover, continuous exposure to FSH stimulation in patients with gonadotropin adenomas has triggered continuous ovulation, causing the ovary to contain both follicular cysts and the corpus luteum, which explains the elevated P levels.

Ovarian enlargement stimulates the peritoneum and the activation mechanism of sOHSS autoreceptors, which increases serum CA125 and makes it difficult to identify benign and malignant ovarian tumours. It is easy to misdiagnose ovarian tumours in the clinic and to advise surgery, but the pathological results are often reported as ovarian simple cysts or follicular cysts, which result in iatrogenic overtreatment. This reminds us that CA125 and serum hormone levels can be used as auxiliary reference indicators such that when only CA125 increases and serum hormone levels remain normal, the possibility of malignant ovarian tumours should still be considered first, and when CA125 and hormone levels change at the same time, attention should be paid to the possibility of sOHSS.

### Diagnosis and Differential Diagnosis

For the diagnosis of FSH-secreting pituitary adenoma, we need to combine the results of immunohistochemistry, serum hormone levels, clinical manifestations and ultrastructural observations. In the diagnosis of sOHSS, we emphasize the importance and necessity of ultrasonography and sex hormone testing. Due to the extremely low incidence of the disease, clinicians are not aware of it, so it is prone to misdiagnosis. When patients with recurrent multiple ovarian cysts with simultaneous elevation of E_2_, PRL, mildly elevated or normal FSH, and decreased LH, a pituitary adenoma is highly suspected. The final diagnosis depends on pathology, immunohistochemistry and the improvement of postoperative symptoms.

Patients with polycystic ovary syndrome(PCOS) are at high risk for iatrogenic ovarian hyperstimulation, and the FSH/LH ratio is usually decreased in PCOS and elevated in subjects with pituitary tumours, so attention should be paid to the diagnosis of these diseases. Due to the long-term hyperestrogen overstimulation of the endometrium, repeated breakthrough bleeding and endometrial atypical hyperplasia often occur. Care should be taken to distinguish sOHSS from ovarian tumours, especially granulosa cell tumours.

### Treatment and Prognosis

At present, there is no standardized treatment for FSH-secreting pituitary adenoma, but such studies are being undertaken. Regarding drug treatment, dopamine agonists have certain therapeutic effects. Previously, there was a case report in which two patients with pituitary adenoma saw a stop in their lactation symptoms after treatment with bromocriptine, their menstruation returned to normal, and natural pregnancy and delivery were successful. However, the literature indicates that after treatment with cabergoline, serum prolactin and oestrogen decreased and ovarian cysts shrank, but pituitary tumours did not shrink (25). The application of gonadotropin-releasing hormone agonist (GnRH-a) has no obvious effect on the treatment of pituitary tumours (2, 26). Our case shows that one month after the injection of triptorelin, the levels of oestradiol and FSH could not be reduced; instead, the ovarian cysts were further enlarged. Meanwhile, GnRH-a may increase the risk of iatrogenic OHSS. The therapeutic effect of GnRH antagonists is currently inconsistent. Some studies have shown that GnRH antagonists treatment can reduce the ovarian volume, but the ovaries increase again after discontinuation of treatment (27). In other studies, the level of FSH did not decrease significantly, but the level of serum E_2_ and the volume of ovary decreased significantly (14). At the same time, its effect on the central pituitary gland may have a direct effect on granulosa cells, inhibiting the activity of aromatase and helping to reduce E_2_ levels and to improve clinical symptoms.

We also found that radiotherapy (gamma knife) has limited therapeutic effect on pituitary tumor, and its recurrence rate is very high, so it can only be used as an auxiliary means of clinical treatment (28). The radical cure for pituitary adenoma is transsphenoidal surgery (9, 29). Of the 37 published cases, 8 patients had spontaneous pregnancy after pituitary tumour resection, but 7 patients exhibited recurrence after surgery, and 3 of them underwent reoperation. Most of the patients’ enlarged ovaries gradually atrophied and returned to a normal size after the removal of adenoma; further, ovarian function was restored, and the patients could return to a normal menstrual cycle (30, 31). However, there is no obvious capsule in this kind of tumour, so it is difficult to grasp the scope of tumour resection and eradicate it, so postoperative recurrence is more common. At the same time, secondary hypophysis and other related complications may occur. Our case also shows that if postoperative patients have menstrual disorders or secondary amenorrhea caused by recurrent pituitary adenoma, then we can consider using assisted reproductive technology to achieve the purpose of pregnancy.

The increase in serum E_2_ concentration and continuous FSH secretion during the follicular phase is a way to monitor oocyte maturation (18, 23); however, oocyte development in the ovary even requires a sufficient amount of LH secretion. Our case also found that due to the lack of LH secretion in such patients, ovulation could not be induced; therefore, we had to administer exogenous hCG to help oocyte maturation and ovulation. This case showed that mature oocytes can be obtained and healthy infants can be delivered even if the serum LH concentration is lower than the conventional level. As shown in a report, mature oocytes are obtained even if controlled ovarian stimulation is initiated in various phases of women’s menstrual cycle. Besides, our case suggests that oocyte maturation could be more flexible than we generally believe.

## Conclusion

In conclusion, sOHSS caused by FSH-secreting pituitary adenoma is easily misdiagnosed and missed in the clinic. When multiple ovarian cysts occur in women of reproductive age, serum E_2_ and PRL levels increase, FSH levels increase or are normal, and LH levels decrease. A detailed and comprehensive examination of the nervous system and sex hormones is very important to exclusion of the diagnosis of FSH-secreting pituitary adenoma and to effectively avoid unnecessary ovarian surgery. Dopamine agonists can control hyperprolactinemia, but no drug has been found to significantly reduce tumour size. GnRH agonists are not recommended for the treatment of this disease, and the current effect of GnRH antagonists is uncertain. The radical treatment of the disease is still mainly pituitary tumour resection. Transsphenoidal surgery is the first option. For patients whose menstrual cycle and ovulation have not returned to normal after surgery or who experience secondary hypomenorrhea caused by excessive tumour resection, IVF-ET should be considered to help them achieve pregnancy.

## Data Availability Statement

The original contributions presented in the study are included in the article/supplementary material. Further inquiries can be directed to the corresponding author.

## Ethics Statement

Written informed consent was obtained from the individual(s) for the publication of any potentially identifiable images or data included in this article.

## Author Contributions

YG developed the conceptual framework for the study. XD conducted the data collection. WZ and XW interpreted the data. XY and ZL edited the figure and tables. XD and WZ drafted the manuscript. YG made major revisions. All authors contributed to the article and approved the submitted version.

## Conflict of Interest

The authors declare that the research was conducted in the absence of any commercial or financial relationships that could be construed as a potential conflict of interest.
